# Nutritional impact of inflammatory bowel diseases on children and adolescents☆

**DOI:** 10.1016/j.rpped.2014.04.008

**Published:** 2014-12

**Authors:** Gilton Marques dos Santos, Luciana Rodrigues Silva, Genoile Oliveira Santana

**Affiliations:** Universidade Federal da Bahia (UFBA), Salvador, BA, Brazil

**Keywords:** Nutritional assessment, Nutritional status, Inflammatory bowel diseases, Child, Adolescent

## Abstract

**OBJECTIVE:**

To perform a sistematiy review of the literature about the nutritional impact of inflammatory bowel diseases in children and adolescents.

**DATA SOURCES:**

A systematic review was performed using PubMed/MEDLINE, LILACS and SciELO databases, with inclusion of articles in Portuguese and in English with original data, that analyzed nutritional aspects of inflammatory bowel diseases in children and adolescents. The initial search used the terms "inflammatory bowel diseases" and "children" or "adolescents" and "nutritional evaluation" or "nutrition deficiency". The selection of studies was initially performed by reading the titles and abstracts. Review studies and those withouth data for pediatric patients were excluded. Subsequently, the full reading of the articles considered relevant was performed.

**RESULTS:**

237 studies were identified, and 12 of them were selected according to the inclusion criteria. None of them was performed in South America. During the analysis of the studies, it was observed that nutritional characteristics of patients with inflammatory bowel disease may be altered; the main reports were related to malnutrition, growth stunting, delayed puberty and vitamin D deficiency.

**CONCLUSION:**

There are nutritional consequences of inflammatory bowel diseases in children and adolescents, mainly growth stunting, slower pubertal development, underweight and vitamin deficiencies. Nutritional impairments were more significant in patients with Crohn's disease; overweight and obesity were more common in patients with ulcerative rectocolitis. A detailed nutritional assessment should be performed periodically in children and adolescents with inflammatory bowel disease.

## Introduction

Inflammatory bowel diseases (IBDs) are represented by ulcerative rectocolitis (URC), Crohn's disease (CD) and indeterminate colitis,^1^ which affect the gastrointestinal tract in different ways, mainly the ileum and other segments of the jejunum, very often the colon in CD and only the colon and/or rectum in URC. These diseases have overlapping clinical and pathological characteristics; however, some have distinct features.^2^^,^ 3

There are several predisposing factors for their onset, such as genetic, environmental, and immunological factors, and an important role is played by the intestinal microbiota imbalance, as well as by changes in mucosal permeability and immune response. The risk factor to be emphasized is the reporting of similar cases in the family, especially in first-degree relatives.^4^^,^ 5 IBDs have a worldwide distribution, with increasing incidence in recent decades in all geographic areas, with two peaks of incidence, one in adolescence and another in adulthood. Children are also affected, and the incidence in this age range is also increasing.^6^

In CD there is transmural inflammation affecting any segment of the gastrointestinal tract, from mouth to anus, especially distal small intestine and colon, while URC is restricted to the mucosa and submucosa and can involve the colon and rectum. Both can have extraintestinal manifestations and variable severity. When clinical, radiological, endoscopic and histopathological criteria are not completely consistent, the disease is classified as indeterminate colitis.^7^^,^ 8

Nutrition is an extremely important aspect for patients with chronic diseases, especially in the pediatric age range. Nutritional problems are common in patients with IBDs, accompanied by clinical exacerbation of disease activity and remission, depending on the activity, the location, extension, severity and presence of complications. Nutritional changes can be identified in 20-85% of patients, especially in patients with CD.^9^^,^ 10

There may be growth delay, malnutrition, specific micronutrient deficiencies, or eventually even overweight and obesity. The determinants of nutritional changes are: reduction of food intake, intestinal malabsorption, gastrointestinal losses due to inflammation, increased nutritional needs due to disease activity, concomitant infections, reduced food intake due to decreased appetite or due to fear of worsening symptoms, immunosuppressive treatment, side effects of medications and even surgical resections, and other systemic complications and involvement of other organs, which may cause weight loss, anemia, anorexia, hypoalbuminemia, negative nitrogen balance and nutrient and vitamin deficiencies.^11^^-^ 13

IBDs can cause manifestations of the digestive tract as well as extradigestive ones. These conditions also determine several psychosocial and economic problems, missed days at work and school, depression, change in body image and low self-esteem, sexuality and socialization difficulties, feeding difficulties, fear to leave the house and not finding a public restroom available, among others, which limit the quality of life. Patients also report feeding problems, a fact that contributes to the worsening of the nutritional impairment, to the development of anemia, stunted growth and systemic complications.^14^

Nutritional alterations represent public health problems, which affect all strata of society. IBDs can aggravate nutritional deficiencies, causing malnutrition, but in some cases they may be associated with overweight as a result of drugs and aspects of binge eating. Inadequate nutritional status is a poor prognosis and may influence treatment response, as well as morbidity and mortality.^15^^,^ 16

Nutritional assessment is of great importance for all pediatric patients, especially those with IBDs, allowing early identification of nutritional alterations, and it can be performed with anthropometric data, biochemical indices, sophisticated tests, and even through nutritional survey.^18^^,^ 19 One of the most used methods is the assessment of Body Mass Index (BMI), calculated by dividing the patient's weight by the squared height. Based on the BMI vs. age charts and the z score, one can classify the nutritional status of children.^20^^,^ 21 Adequate nutritional status, growth and development are key aspects in pediatric patients from the neonatal period to adolescence, particularly in patients with chronic diseases.

There is a scarcity of studies on nutritional assessment in pediatric patients with IBDs,^3^ and there have been no reports in children and adolescents in Brazil. This study aimed to perform a systematic review on the nutritional implications of the presence of IBDs in children and adolescents.

## Method

To develop this study, a systematic review was performed in PubMed/ MEDLINE, SciELO and LILACS databases to identify the literature published from January 2006 to January 2013, using the following terms: (Nutritional assessment OR nutritional disorders OR body compositions) AND (Inflammatory bowel diseases OR Crohn disease OR ulcerative disease) AND (Children OR pediatrics OR adolescents). Only studies published in Portuguese and English, with original data that analyzed dietary habits of children and adolescents with IBDs were included. Review studies and those with no results for pediatric patients were excluded. 

**Figure 1:**
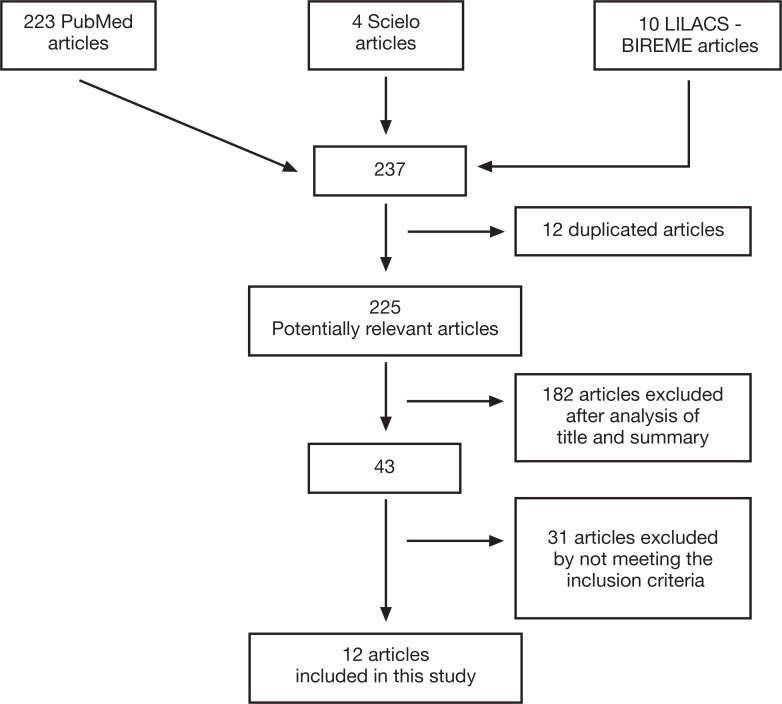
Flowchart of the systematic review of inflammatory bowel diseases in the pediatric age range and nutritional aspects

Study selection was initially performed by reading the titles and abstracts of articles. In the second phase, the reading of the methods section was performed, and in the third phase, the reading of the full texts was carried out. The non-relevant articles were discarded (Fig. 1). After analyzing and interpreting the articles, the information was organized in thematic groups. As this is a systematic review, it was not necessary to submit the study to the ethics committee for approval.

## Results

After the search for published articles on the topic, the review identified 237 studies published during the defined period on the nutritional aspects of children and adolescents with IBDs. A total of 223 articles were found in PubMed, 10 in LILACS and 4 in SciELO. After exclusions, 12 articles were selected for final assessment, and they comprised this systematic review (Fig. 1). The selected studies were carried out in four countries: United States, United Kingdom, Australia and Pakistan, with nine of them having been carried out in the United States. There were no studies carried out in Brazil. The list is shown in Table 1.

**Table 1:**
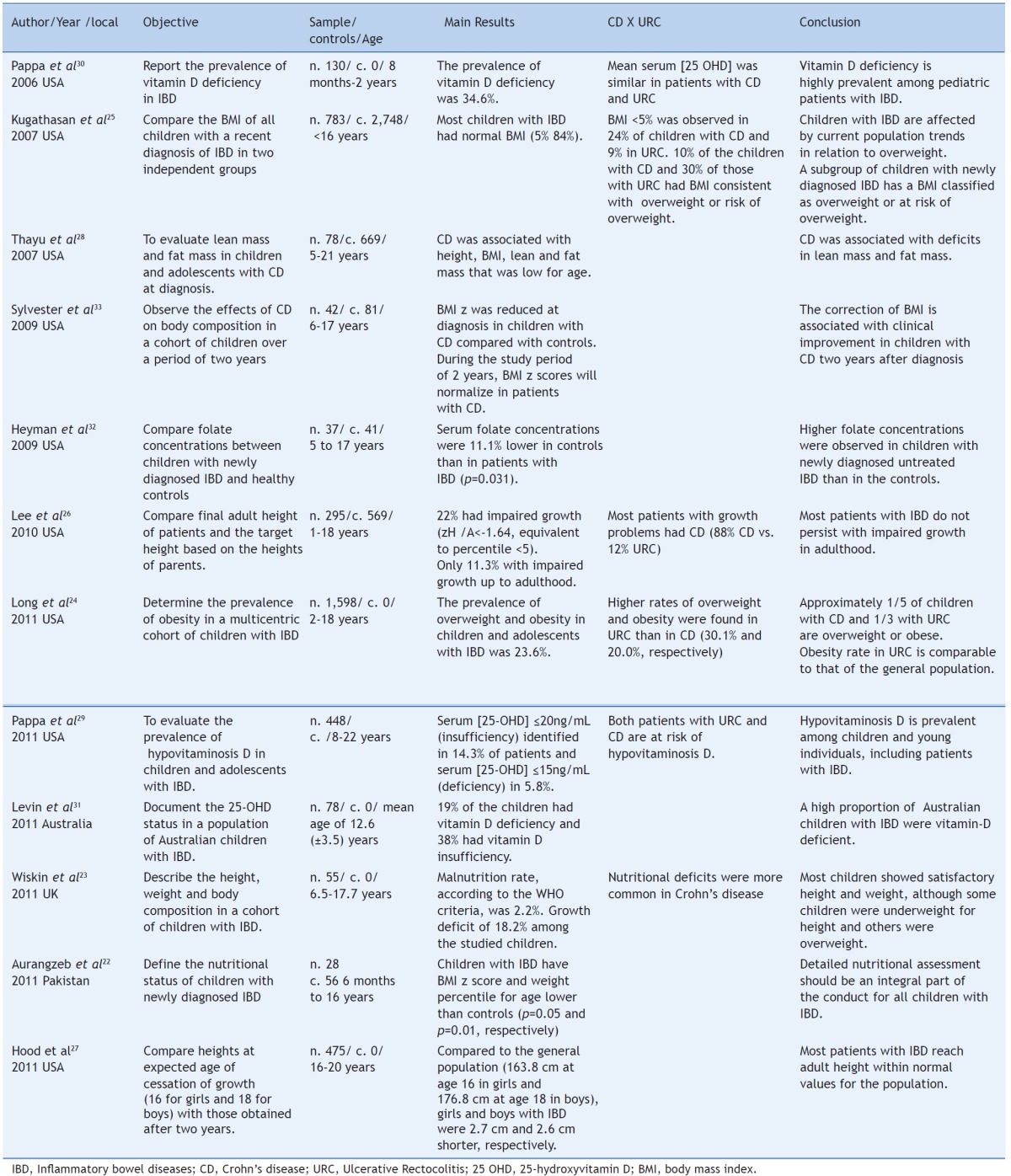
Characteristics and description of studies on nutritional aspects in children and adolescents with inflammatory bowel diseases included in the systematic review.

### Nutritional Characteristics in IBDs

The analyzed studies showed that pediatric IBD patients, especially those with CD, have alterations in nutritional status, and malnutrition is frequently reported. Aurangzeb *et al* 22 studied 28 children with IBD and compared them with healthy children. The first showed lower BMI z score and lower weight for age (*p*=0.05 and *p*=0.01, respectively), and lower levels of leptin, suggesting malnutrition. Almost half of these children were diagnosed in the age group of 9-12 years (prepubertal or pubertal), with the presence of nutritional impairment in an important phase of growth and development. Wiskin *et al* 23 assessed 55 children with IBD and found a malnutrition rate of 2.2%, according to the WHO criteria.

There is a tendency for underweight in patients with IBDs, but studies have shown some changes in that profile, such as an increase in the number of overweight or obese individuals in recent years, especially in those with URC. Long *et al* 24 assessed the prevalence and epidemiology of obesity in 1,598 children and adolescents aged 2-18 years with IBD and found that 23.6% were obese or overweight. The study suggests that, with the advent of treatments for IBDs, malnutrition and underweight may cease to be a marker of IBD severity, with children showing high rates of overweight and obesity, as well as the general population. Kugathasan *et al* 25 assessed 783 patients with IBD and showed that the majority (68%) was within the normal weight range, and that of the newly diagnosed patients with IBDs, 9-34%, depending on the type, were overweight - at a lower rate (7-24%), they had underweight or underweight risk.

Growth delay is common in patients with a diagnosis of IBD in childhood and this may be the only symptom. Wiskin *et al*, 23 in 2011, observed growth delay in 18.2% of the children studied. Lee *et al*,^26^ also in 2011, assessed 295 patients with IBD diagnosed between the ages of 1-18 years in a prospective cohort study and found that 22% had growth problems, according to the z score of height for age. The same authors observed a growth curve with a slight shift to the left in patients with IBD, while 11.3% of these patients had impaired final adult height. The same authors also found that patients with growth problems achieve lower mean final target height than healthy individuals.

Hood *et al*,^27^ in 2011, assessed 475 men and women with IBD aged 16, 18 and 20 years to evaluate the final adult height and found no significant differences between the height of patients with IBD and that of healthy individuals, despite the identified growth problems. These authors emphasize the importance of identifying patients with early growth retardation. Aurangzeb *et al* 22 and Hood *et al* 27 reported that those diagnosed in the prepubertal or pubertal stages are more vulnerable to pubertal and growth delay, and consequently, to a compromised final height, especially if the diagnosis occurs before Tanner stages 1 and 2. Thayu *et al* 28 analyzed 78 patients with CD and 669 controls aged 5 to 21 years and found older age for the same Tanner stage in CD patients, demonstrating that, in association with growth retardation, children and adolescents with IBDs often have pubertal delay.

Nutrient and vitamin deficiencies are reported, mainly vitamin D deficiency. Pappa *et al*,^29^ in 2011, assessed 448 patients with IBD and showed that hypovitaminosis D is prevalent in children and adolescents. At least one measurement of serum 25-hydroxyvitamin D (25-OHD) was reported in this study and classified as optimal (>32ng/mL), suboptimal (≤2ng/mL), insufficient (≤20ng/mL) and deficient (≤15ng/mL) levels. Serum 25-OHD levels less than optimal were reported in 58.5% of patients, and insufficient or deficient levels in 20.1%.

The association of vitamin D deficiency with risk factors is similar to that of the general population, when considering geographic areas with little exposure to sunlight at certain seasons of the year (winter and spring), dark skin, high BMI and lack of vitamin D supplementation, in addition to malabsorption of calcium and vitamin D due to intestinal lesion and chronic use of medications, especially corticosteroids. Pappa *et al*,^30^ in 2006, analyzed the status of vitamin D in children with IBD, in a cross-sectional study with 130 patients, and the prevalence of deficiency (≤15ng/mL of 25-OHD) and severe deficiency (≤15ng/mL of 25-OHD) of vitamin D was present in 34.6% and 10.8%, respectively. 

Pappa *et al*,^29^ in 2011, observed lower concentrations of 25-OHD in young patients with active disease; in severe disease; in those not receiving vitamin D supplements; in patients with some nutritional impairment; in patients with newly diagnosed disease, and in those with more extensive disease. 

Levin *et al* 31 analyzed serum 25-OHD levels in 78 children and classified them as: severe deficiency (<30nmol/L); moderate deficiency (<51nmol/L) and insufficiency (between 51 and 70 nmol/L), and found 57.5% of individuals with serum 25-OHD levels lower than the ideal, and 19.2% with moderate or severe deficiency. The authors concluded that newly diagnosed patients had higher mean serum levels of 25-OHD when compared to those with long-term disease, and that patients with deficiency had longer time of exposure to corticosteroids. Other nutritional analyses were performed in the selected studies.

Aurangzeb *et al*,^22^ in 2011, found mean lower levels of serum leptin in children with IBD when compared to controls, 2.4 and 5.2pg/mL, respectively. Heyman *et al*,^32^ in 2009, found that folate levels in pediatric patients with untreated IBD was higher than that obtained in healthy controls, although folic acid intake was higher in the controls. They also observed that anemia in patients with IBD does not depend on folate levels. Sylvester *et al*,^33^ in 2009, found that lean mass and bone mineralization were significantly lower in patients with IBD, when compared to controls.

### Nutritional assessment in pediatric patients with IBDs

Of the 12 selected studies, almost all patients had data on height, weight and BMI, expressed directly or as z scores. Aurangzeb *et al* 22 observed that the BMI z score and weight-for-age percentile in IBD patients were significantly lower than in controls (*p*<0.05 and *p*<0.01, respectively). Heyman *et al* 32 found that the mean BMI in patients with IBDs was lower than in controls, but the difference was not significant (18.7 and 20.1kg/m² respectively, *p*=0.189). Thayu *et al* 28 reported that patients with CD had significantly lower height and z scores of BMI than the control children (*p*<0.001). 

Pappa *et al*,^29^^,^ 30 in two studies (2006 and 2011), analyzed height, age and BMI (z scores) and observed that high BMI is a risk factor for low 25-OHD levels (negative association). Kugathasan *et al* 25 followed the weight and height for two years and observed that BMI increased significantly during the period, reaching normal rates when compared to controls, but lean mass values remained lower than that of controls, suggesting that BMI alone is not sufficient for nutritional assessment in these patients, and the association with other methods is necessary. Kugathasan *et al* 25 showed that children with normal BMI can have IBDs, especially URC.

### Crohn's Disease X Ulcerative Rectocolitis in Pediatric Patients

When comparing types of IBDs, there is a greater prevalence of nutritional impairment in patients with CD when compared to those with URC. Pappa *et al* 30 observed that patients with CD have significantly lower mean z score values ​​of weight, height, and BMI when compared to those with URC (*p*=0.001, *p*=0.002 and *p*=0.03), as well as albumin values (*p*=0.02), suggesting greater severity in CD.

When assessing excess weight and obesity in children and adolescents, it was observed that despite the tendency for increased risk of obesity and overweight in IBD, CD patients had lower values ​​when compared to those with URC. Long *et al* 24 found that the prevalence of obesity or overweight was 20% in patients with CD *versus* 30% of those with URC. CD patients with excess weight had higher rates of complications, such as IBD-related surgeries. Patients with URC who use corticosteroids have a greater association with excess weight.

Kugathasan *et al* 25 found a prevalence of excess weight or obesity of approximately 10% in CD, and 20% to 30% in URC. The authors also observed that the BMI value at diagnosis depends on the type of disease, with less excess weight and a higher rate of malnutrition in patients with CD. Lee *et al*,^26^ when assessing the linear growth of children and adolescents with IBD, found that growth delay is significantly more prevalent in CD than in URC, with growth problems in 88% of patients with CD and only 12% in those with URC. Unlike the previous observations, some studies found a higher prevalence of growth deficit in patients with URC. 

Pappa *et al* 29 observed that the mean concentration of serum 25-OHD was 8.2% lower in patients with URC, when compared to those with CD, but without statistical significance (*p*=0.12). Levin *et al* 31 observed significantly lower mean levels of 25-OHD (*p*=0.02) in patients with URC, when compared to CD patients with serum levels of 54.4nmol/L and 73.1nmol/L. In the study by Heyman *et al*,^32^ the mean folate level was similar in patients with CD and URC.

## Discussion

The assessment of the nutritional status of children and adolescents with IBDs is crucial in the clinical follow-up, to ensure normal growth and development, to define appropriate drug and nutritional therapy, and promote a better quality of life. Another aspect is the need to prevent prolonged corticosteroid therapy, due to the consequent growth impairment.^34^^,^ 35 It is essential to identify nutritional deficiencies that usually manifest as decreased weight for height and/or height for age, as well as specific nutritional deficiencies in children and adolescents with IBDs, and pubertal delay in adolescents. 

Therefore, the nutritional assessment can guide the dietary therapy and adequate supplementation and provide an early correction of nutrient deficiencies, helping to reduce disease activity and minimize symptoms.^36^^,^ 37

The studies included in this review demonstrate that children and adolescents with IBDs, especially those with CD, may have nutritional impairment due to several factors, such as reduced food intake, malabsorption, high intestinal losses, increased energy expenditure due to chronic inflammation and medications.^38^^,^ 39 These effects can lead to problems such as malnutrition, underweight, growth and developmental delay, delayed onset of pubertal characteristics, anemia, osteopenia and osteoporosis, micro and macronutrient deficiencies, in addition to significant psychological problems related to self-image and social interaction with peers, difficulties in the onset of sexual activity and depression.^40^

Other studies have shown that there is greater nutritional impairment in CD patients when compared to those with URC, as a consequence of the greater severity and extent of the affected area in the digestive tract. CD usually affects the small intestine segments responsible for absorption, as well as frequently affecting the colon and other parts of the digestive tract.^35^^-^ 37 Not only the extent of the involvement, but also the severity, presentation, extraintestinal manifestations and comorbidities that may impair the nutritional status should be taken into account.^34^^,^ 41

If not identified and treated, malnutrition in pediatric patients may result in severe health problems, worsening prognosis and consequent reduction in immune competence, increased infections, growth and development delay, social problems and higher rates of comorbidities. Stunted growth and pubertal delay are significantly important manifestations in pediatric patients and may be the first clinical findings of this condition, an aspect observed in several studies^35^^-^ 37 emphasizing the growth delay, which was reported in 18 to 22% of patients.

Several studies have shown high rates of growth retardation, often preceding the intestinal manifestations, ranging between 5% and 88%, with CD patients experimenting higher rates of nutritional impairment when compared to patients with URC. In association with growth retardation, impairment of pubertal development is observed in 20-30% of patients, mainly in those with CD^38^^,^ 41 ^,^ 42 of multifactorial etiology.^35^^,^ 36

Other explanations for growth delay are associated with the use of medications, especially corticosteroids and hormonal disturbances caused by both the direct effects of the inflammatory processes and pubertal delay. Children with an early diagnosis of IBD in prepubertal or pubertal stages deserve special attention regarding linear growth.^35^^,^ 37 ^,^ 42

Another aspect is the change in the overweight or obesity profile in the pediatric population with IBDs, with a tendency to the increase in the number of patients with overweight or obesity, ranging from 9-34%, depending on the disease subtype, with a higher prevalence in patients with URC. In a study carried out in the United States, high BMI was found in 20-30% of patients, with the highest values ​​in patients with ulcerative rectocolitis.^41^ This increase in rates of overweight and obesity in pediatric patients with IBDs may be explained, in part, by the epidemic of metabolic syndrome that occurs worldwide, due to changes in eating habits.

Studies have shown that deficiencies of minerals, trace elements and vitamins are common in patients with IBDs, with vitamin D deficiency being the most common, reaching 60% in some cases.^41^ These several deficiencies reflect chronic blood loss, chronic diarrhea or impairment of specific absorption sites, in addition to malabsorption due to the extent of inflammation, or surgical resections.^34^ In some studies, the most common deficiency was that of vitamin D in patients with URC, when compared to those with CD, differing from publications in the literature that showed a higher prevalence in patients with CD.^38^^,^ 40

## Conclusions

A limited number of articles was found, and no studies on this subject have been carried out in South America. Nutritional alterations of multifactorial etiology can be found in inflammatory bowel diseases in children, especially growth and pubertal delay, underweight and vitamin deficiencies.

The reported nutritional impairments were more significant in patients with Crohn's disease, and overweight and obesity more common in ulcerative rectocolitis. A detailed nutritional assessment should be performed periodically in all children and adolescents with inflammatory bowel diseases, combined with different methods and multidisciplinary resources. More studies are needed in the pediatric age range with larger sample sizes.
