# Supplementary material for: Nutritional impact of inflammatory bowel diseases on children and adolescents
Source: Rev Paul Pediatr. 2014 Dec;32(4):403–11. doi: 10.1016/j.rpped.2014.04.008 (PMC4311796; doi:10.1016/j.rpped.2014.04.008)
Supplement: Translation

# Translation: Repercussões nutricionais em crianças e adolescentes na presença de doenças inflamatórias intestinais☆

**Authors:** Gilton Marques dos Santos, Luciana Rodrigues Silva, Genoile Oliveira Santana

**Affiliations:** a Universidade Federal da Bahia (UFBA), Salvador, BA, Brasil

**Keywords:** Avaliação nutricional, Estado nutricional, Doenças inflamatórias intestinais, Criança, Adolescente

## Abstract

**OBJETIVO:**

Realizar revisão sistemática de literatura sobre repercussões nutricionais em crianças e adolescentes na presença de doenças inflamatórias intestinais.

**FONTES DE DADOS:**

Realizada revisão sistemática utilizando as bases de dados PubMed/MEDLINE, LILACS e SciELO, com inclusão de artigos em língua portuguesa e inglesa com dados originais que analisaram aspectos nutricionais de crianças ou adolescentes com doenças inflamatórias intestinais. Na busca inicial, utilizaram-se os termos *"inflammatory bowel diseases" and "children" or "adolescents" and "nutritional evaluation" or "nutrition deficiency"*. A seleção de estudos foi feita, inicialmente, por meio da leitura dos títulos e resumos. Foram excluídos estudos de revisão e sem resultados para faixa pediátrica. Em um segundo momento, foi realizada leitura completa dos artigos considerados relevantes.

**SÍNTESE DE DADOS:**

Foram identificados 237 estudos - desses, 12 foram selecionados de acordo com os critérios de inclusão, não havendo nenhum na América do Sul. Na análise dos artigos, foi observado que características nutricionais em pacientes com doenças inflamatórias intestinais podem estar alteradas, sendo relatados principalmente desnutrição, retardo de crescimento e puberdade e deficiência de vitamina D.

**CONCLUSÃO:**

Há alterações nutricionais nas doenças inflamatórias intestinais em pediatria, ressaltando-se parada no crescimento e desenvolvimento puberal, baixo peso e deficiências vitamínicas. Os comprometimentos nutricionais relatados são mais expressivos nos pacientes portadores de Doença de Crohn, e sobrepeso e obesidade mais frequentes na Retocolite Ulcerativa. A avaliação nutricional detalhada deve ser realizada periodicamente em todas crianças e adolescentes portadores de doenças inflamatórias intestinais.

## Introdução

As doenças inflamatórias intestinais (DII) são representadas pela retocolite ulcerativa (RCU), doença de Crohn (DC) e colites indeterminadas,^1^ que comprometem de modo diverso o trato gastrointestinal, sobretudo íleo e outros segmentos do jejuno, muitas vezes o colón na DC e apenas cólon e/ou reto na RCU. São condições com características clínicas e patológicas que se sobrepõem, no entanto têm algumas particularidades distintas.^2^^,^ 3

Há vários fatores predisponentes para o seu desencadeamento, tais como os genéticos, ambientais, imunológicos e um papel importante representado pelo desequilíbrio da microbiota intestinal, alterações na permeabilidade e na resposta imune da mucosa. O fator de risco a ser ressaltado é o relato de casos semelhantes na família, sobretudo em parentes de primeiro grau.^4^^,^ 5 As DII apresentam uma distribuição mundial, com incidência crescente nas ultimas décadas em todas as áreas geográficas, com dois picos de incidência, um na adolescência e outro na vida adulta. As crianças também são acometidas e a incidência nesta faixa etária também vem aumentando.^6^

Na DC há inflamação transmural, afetando qualquer segmento do trato gastrointestinal, da boca ao ânus, principalmente intestino delgado distal e colón, enquanto a RCU fica restrita à mucosa e submucosa, podendo acometer o colón e o reto. Ambas podem ter manifestações extraintestinais e gravidade variável. Quando os critérios clínicos, radiológicos, endoscópicos e histopatológicos não são completamente consistentes, a doença é classificada como Colite Indeterminada.^7^^,^ 8

A nutrição representa um aspecto de extrema importância para portadores de doenças crônicas, sobretudo na faixa etária pediátrica. Problemas nutricionais são comuns em pacientes portadores de DII acompanhada de quadros com exacerbação de atividade inflamatória e remissão, a depender da atividade, da localização, da extensão, da gravidade e da presença de complicações. Alterações nutricionais podem ser identificadas em 20 a 85% dos pacientes, em especial naqueles portadores de DC.^9^^,^ 10 Pode haver retardo no crescimento, desnutrição, deficiências específicas de micronutrientes ou, eventualmente, até sobrepeso e obesidade. Como determinantes das alterações nutricionais estão: redução da ingestão alimentar, má absorção intestinal, perdas gastrointestinais decorrentes da inflamação, necessidade nutricional aumentada pela atividade da doença, infecções concomitantes, diminuição da ingestão pela diminuição do apetite ou devido ao medo da piora dos sintomas, tratamento imunossupressor, efeitos colaterais das medicações e até ressecções cirúrgicas e outras complicações sistêmicas e acometimento de outros órgãos, que podem determinar perda de peso, anemia, anorexia, hipoalbuminemia, balanço nitrogenado negativo e deficiências de nutrientes e vitaminas.^11^^-^ 13

As DII podem causar manifestações do trato digestório e extradigestivas. Estas condições também determinam vários problemas psicossociais e econômicos, falta ao trabalho e escola, depressão, alteração de imagem corporal e baixa autoestima, dificuldades de socialização e sexualidade, dificuldades com a alimentação, medo de sair e medo de não encontrar banheiro público disponível, entre outros, que limitam a qualidade de vida. Pacientes relatam problemas com alimentação, fato que contribui para o agravamento do comprometimento nutricional, para desenvolvimento de anemia, parada no crescimento, além de complicações sistêmicas.^14^ Alterações nutricionais representam problemas de saúde pública, que afetam todas as camadas da sociedade. As DII podem agravar o quadro de carência nutricional, determinando desnutrição, mas, em alguns casos, podem se associar a sobrepeso, em decorrência dos medicamentos e aspectos de compulsão alimentar. O estado nutricional inadequado é um fator prognóstico ruim e pode influenciar a resposta ao tratamento e a morbidade e mortalidade.^15^^,^ 16

A avaliação nutricional é de grande importância para todos pacientes pediátricos, especialmente os portadores das DII, permitindo reconhecimento precoce das alterações nutricionais e pode ser realizada com dados antropométricos, índices bioquímicos, testes sofisticados e, até, por meio de inquérito nutricional.^18^^,^ 19 Um dos métodos mais utilizados é a avaliação do Índice de Massa Corporal (IMC), calculado através da divisão do peso do paciente pela segunda potência de sua altura. Baseando-se nos gráficos IMC x Idade e a partir do escore z, pode-se classificar o estado nutricional das crianças.^20^^,^ 21 Estado nutricional, crescimento e desenvolvimento adequados são aspectos fundamentais na faixa etária pediátrica desde o período neonatal até a adolescência, sobretudo em pacientes com doenças crônicas. 

Há uma grande carência de estudos sobre avaliação nutricional em pediatria nos portadores de DII^3^ e, no Brasil, não há relatos em crianças e adolescentes. O presente estudo teve como objetivo realizar revisão sistemática sobre as repercussões nutricionais da presença de DII em crianças e adolescentes. 

## Método

Para desenvolver este estudo, foi realizada revisão sistemática nas bases de dados PUBMED/MEDLINE, Scielo e LILACS, visando identificar a literartura publicada, no período de janeiro de 2006 a janeiro de 2013, os termos de busca: (Nutritional assessment OR nutritional disorders OR body compositions) AND (Inflammatory bowel diseases OR Crohn disease OR ulcerative disease) AND (Children OR pediatrics OR adolescents). Foram incluídos apenas estudos em língua portuguesa e inglesa com dados originais que analisaram aspectos nutricionais em crianças ou adolescentes portadores de DII. Foram excluídos estudos de revisão e sem resultados para a faixa pediátrica. A seleção de estudos foi realizada inicialmente por meio da leitura dos títulos e resumos dos artigos. No segundo momento, foi realizada a leitura dos métodos, e no terceiro momento, foi feita a leitura completa dos artigos. Os artigos não relevantes foram descartados (fig. 1). Após análise e interpretação dos artigos, as informações foram organizadas em grupos temáticos. Por se tratar de revisão sistemática, não foi necessária submissão ao comitê de ética. 

**Figura 1:** Diagrama de fluxo da revisão sistemática sobre doenças inflamatórias intestinais em pediatria e aspectos nutricionais

## Resultados

Após a busca de estudos na literatura sobre o tema, o processo de revisão identificou 237 estudos publicados no período sobre aspectos nutricionais de crianças e adolescentes com DII. Foram encontrados 223 artigos no PUBMED, 10 no LILACS e 4 no Scielo. Após exclusões, 12 artigos foram selecionados para avaliaçõ final e compuseram esta revisão sistemática (fig. 1). Os estudos selecionados se originaram de quatro países Estados Unidos, Reino Unido, Austrália e Paquistão, sendo nove dos Estados Unidos. Não foram encontrados estudos brasileiros. A lista pode ser vista na tabela 1. 

**Tabela 1:** Características e descrição dos estudos sobre aspectos nutricionais em crianças e adolescentes com doenças inflamatórias intestinais incluídas na revisão sistemática.

### Características Nutricionais em DII

Os estudos analisados demonstraram que pacientes pediátricos com DII, principalmente aqueles portadores de DC, apresentam alteração no estado nutricional e a desnutrição é frequentemente relatada. Aurangzeb *et al*22 analisaram 28 crianças portadoras de DII e compararam com crianças sadias. As primeiras mostraram menor z score de IMC e menor peso para idade (*p*=0,05 e *p*=0,01, respectivamente), além de menores níveis de leptina, sugerindo desnutrição. Quase metade dessas crianças foi diagnosticada na faixa etária de 9 a 12 anos (pré-púbere ou púbere), com presença de comprometimento nutricional numa importante fase de crescimento e desenvolvimento. Wiskin *et al* 23 analisaram 55 crianças com DII e encontraram taxa de desnutrição de 2,2%, segundo critérios da OMS.

Há uma propensão para o baixo peso em pacientes com DII, mas os estudos mostraram algumas mudanças nesse perfil, como aumento nos ultimos anos do número de portadores de sobrepeso ou obesidade, principalmente naqueles com RCU. Long *et al* 24 analisaram a prevalência e a epidemiologia da obesidade em 1598 crianças e adolescentes de 2 a 18 anos portadores de DII e constataram que 23,6% estavam com obesidade ou sobrepeso. O estudo sugere que, com o advento dos tratamentos para DII, a desnutrição e o baixo peso podem deixar de ser um marcador de gravidade das DII, passando as crianças a terem altas taxas de sobrepeso e obesidade, assim como a população geral. Kugathasan *et al* 25 analisaram 783 pacientes com DII e demonstraram que a maioria (68%) está na faixa de peso normal e que, dos pacientes recém-diagnosticados com DII, 9 a 34%, a depender do tipo, apresentam sobrepeso e uma taxa menor (7 a 24%), tinha baixo peso ou risco de baixo peso. 

O retardo de crescimento é comum em pacientes com diagnóstico de DII na infância e este pode ser o único sintoma. Wiskin *et al*,^23^ em 2011, encontraram déficit de crescimento em 18,2% das crianças estudadas. Lee *et al.*,^26^ também em 2011, analisaram 295 pacientes com DII diagnosticados entre 1 a 18 anos em uma coorte prospectiva e encontraram 22% com problemas no crescimento, segundo eo score z de altura para idade. Os mesmos autores observaram, entre os portadores de DII, uma curva de crescimento com um discreto desvio para esquerda, sendo que 11,3% desses pacientes tiveram comprometimento da altura adulta final. Os mesmos autores encontraram ainda que pacientes com problemas de crescimento alcançam altura média final alvo menor que indivíduos sadios. Hood *et al*,^27^ em 2011, analisaram 475 homens e mulheres portadoras de DII com 16, 18 e 20 anos para avaliarem a altura adulta final e não encontraram diferenças significativas entre a altura de portadores de DII e indivíduos saudaveis, apesar dos problemas de crescimento identificados. Estes autores reforçam importância de identificar pacientes com retardo de crescimento precocemente. Aurangzeb *et al* 22 e Hood *et al* 27 relataram que aqueles diagnosticadas nas fases prépúbere ou púbere são mais vulneráveis ao atraso puberal e de crescimento e, consequentemente, ao comprometimento da altura final, principalmente se o diagnóstico acontece antes dos estágios 1 e 2 de Tanner. Thayu *et al* 28 analisaram 78 pacientes com DC e 669 controles entre 5 e 21 anos e encontraram, em pacientes com DC, idade maior para um mesmo estágio de Tanner, demonstrando que, em associação com retardo de crescimento, crianças e adolescentes com DII frequentemente apresentam atraso puberal. 

Deficiências de nutrientes e vitaminas são relatadas, principalmente a deficiência de vitamina D. Pappa *et al*,^29^ em 2011, analisaram 448 pacientes com DII e mostraram que a hipovitaminose D é prevalente em crianças e adolescentes. Pelo menos uma medida da concentração sérica de 25 hidroxivitamina D (25OHD) foi relatada nesse estudo e classificada em: concentração ideal (>32ng/mL), abaixo do ideal (≤2ng/mL), insuficiente (≤20ng/mL) e concentração deficiente (≤15ng/mL). Foram descritos níveis séricos de 25 OHD abaixo do ideal em 58,5% dos pacientes e níveis insuficientes ou deficientes em 20,1%. A associação de deficiência de vitamina D com fatores de risco é semelhante à da população geral, quando se consideram áreas geográficas com pouca exposição à luz solar em certas estações do ano (inverno e primavera), pele escura, IMC alto, falta de suplementação de vitamina D, além de má absorção de cálcio e vitamina D por lesão intestinal e uso crônico de medicamentos, especialmente corticosteroides. Pappa *et al*,^30^ em 2006, analisaram o estado de vitamina D em crianças com DII, em um estudo transversal com 130 pacientes, sendo a prevalência de deficiência (≤15ng/mL de 25 OHD) e de deficiência grave (≤15 ng/mL de 25 OHD) de vitamina D presente em 34,6% e 10,8%, respectivamente. Pappa *et al*,^29^ em 2011, observaram concentrações de 25 OHD mais baixas nos pacientes jovens com doença ativa, na doença grave, naqueles que não tomavam suplementos de vitamina D, nos pacientes com algum comprometimento nutricional, com diagnóstico recente e naqueles com maior extensão da doença. Levin *et al* 31 analisaram concentração sérica de 25 OHD em 78 crianças e classificaram em: deficiência grave (<30 nmol/L); deficiência moderada (<51nmol/L) e insuficiência (entre 51 e 70nmol/L), tendo encontrado 57,5% com níveis séricos de 25 OHD abaixo do ideal e 19,2% com deficiência moderada ou grave. Os autores concluiram que pacientes recém-diagnosticados tinham nível sérico médio de 25 OHD maior do que aqueles com doença de longa duração e que pacientes com deficiência tinham maior tempo de exposição a corticosteroides.Outras análises nutricionais foram feitas nos estudos selecionados. Aurangzeb *et al*,^22^ em 2011, encontraram nível médio de leptina sérica menor em crianças com DII, comparadas relação aos pacientes controles, sendo os valores de 2,4 e 5,2pg/mL, respectivamente. Heyman *et al*,^32^ em 2009, observaram que a concentração de folato em pacientes pediátricos com DII e não tratados foi maior do que a obtida em controles saudáveis, apesar da ingestão de acido fólico ter sido maior nos controles. Observaram, também, que a anemia em pacientes com DII é independente da concentração de folato. Sylvester *et al* 33 em 2009, observaram que a massa magra e a mineralização óssea foram significativamente menores em pacientes com DII, quando comparados aos controles. 

### Avaliação nutricional em doença inflamatória intestinal em pediatria 

Dos 12 estudos selecionados, quase todos tinham dados de altura, peso e IMC, expressos de forma direta ou em z escores. Aurangzeb *et al* 22 observaram que o z escore do IMC e o peso-por-idade em percentil nos pacientes de DII são significantemente menores do que nos controles (*p*<0,05 e *p*< 0,01, respectivamente). Heyman *et al* 32 verificaram que o IMC médio nos portadores de DII é inferior aos controles, porém a diferença não é significante (18,7 e 20,1Kg/m² respectivamente; *p*=0,189). Thayu *et al* 28 relataram que pacientes com DC tinham estatura e z escore de IMC significativamente menores do que as crianças controles (*p*<0,001). Pappa *et al*,^29^^,^ 30 em dois estudos (2006 e 2011), analisaram altura, idade e IMC (z escores) e observaram que o IMC elevado é fator de risco para baixo nível de 25 OHD (associação negativa). Kugathasan *et al* 25 acompanharam o peso e a altura durante dois anos e notaram que o IMC aumentou significantemente durante o período, alcançando taxas normais, comparando-se com os controles, porém os valores de massa magra permaneceram inferiores aos controles, sugerindo que o IMC isolado não é suficiente para a avaliação nutricional nestes pacientes e necessita da associação com outros métodos. Kugathasan *et al* 25 demonstraram que crianças com IMC normal podem ter DII, principalmente RCU.

### Doença de Crohn X Retocolite Ulcerativa em Pediatria

Quando comparados os tipos de DII, nota-se maior prevalência de comprometimento nutricional nos pacientes com DC em relação àqueles com RCU. Pappa *et al*30 observaram que pacientes com DC tem valores médios de z escore de peso, altura e IMC significantemente inferiores, quando comparados com os de RCU (*p*=0,001, *p*=0,002 e *p*=0,03), assim como valores de albumina (*p*=0,02), sugerindo maior gravidade na DC. 

Ao estudar o sobrepeso e a obesidade em crianças e adolescentes, notou-se que, apesar da propensão para o aumento dos riscos desses na DII, pacientes com DC apresentaram valores inferiores em relação àqueles com RCU. Long *et al* 24 observaram que a prevalência de obesidade ou sobrepeso foi de 20% nos pacientes com DC versus 30% daqueles com RCU. Portadores de DC com excesso de peso experimentaram maiores taxas de complicações, tais como cirurgias relacionadas à DII. Pacientes com RCU que usam corticosteroides experimentam maior associação com excesso de peso. Kugathasan *et al* 25 encontraram prevalências de sobrepeso ou de obesidade, cerca de 10% em DC e 20 a 30% em RCU. Esse autor observou, também, que o valor de IMC no momento do diagnóstico depende do tipo de doença, havendo menos sobrepeso e maior taxa de desnutrição nos pacientes com DC. Lee *et al*,^26^ ao avaliarem o crescimento linear de crianças e adolescentes com DII, observaram que o retardo de crescimento é significantemente mais prevalente na DC do que na RCU, com problemas de crescimento em 88% dos pacientes com DC e apenas 12% naqueles com RCU. Ao contrário das observações anteriores, alguns estudos encontraram maiores prevalências de déficits em portadores de RCU. Pappa *et al* 29 observaram que a concentração média de 25 OHD sérico foi 8,2% menor nos pacientes com RCU, quando comparados àqueles com DC, porém não houve significância estatística (*p*=0,12). Levin *et al* 31 notaram níveis médios de 25 OHD significantemente inferiores (*p*=0,02) em portadores de RCU, quando comparados aos pacientes com DC, com valores séricos de 54,4 nmol/L e 73,1nmol/L. No estudo de Heyman *et al*,^32^ a concentração média de folato foi semelhante para os pacientes de DC e RCU.

## Discussão

A avaliação do estado nutricional de crianças e adolescentes portadores de DII é fundamental no acompanhamento clínico para garantir crescimento e desenvolvimento normais, estabelecer terapia medicamentosa e nutricional adequadas e favorecer uma melhor qualidade de vida. Outro aspecto é a necessidade de evitar a corticoterapia prolongada, pelo comprometimento do crescimento.^34^^,^ 35 É essencial identificar deficiências nutricionais que normalmente manifestam-se por meio da diminuição de peso para altura e/ou altura para idade, bem como deficiências nutricionais específicas em crianças e adolescentes portadores de DII, assim como o atraso nas características pubertárias nos adolescentes. Nesse sentido, a avaliação nutricional pode nortear a terapia dietética e a suplementação adequada e corrigir precocemente deficiências de nutrientes, contribuindo para diminuir a atividade da doença e minimizar os sintomas.^36^^,^ 37

Os estudos aqui analisados demonstram que crianças e adolescentes com DII, principalmente aqueles portadores de DC, podem apresentar comprometimento no estado nutricional devido a vários fatores, tais como redução da ingestão alimentar, má absorção, perdas intestinais elevadas, gasto energético aumentado pela inflamação crônica e medicamentos.^38^^,^ 39 Estas repercussões podem gerar problemas como desnutrição, baixo peso, retardo no crescimento e desenvolvimento, retardo no aparecimento dos caracteres puberais, anemia, osteopenia e osteoporose, deficiências de micro e macronutrientes, além de problemas psicológicos significativos relacionados com a autoimagem e a interação social com seus pares, dificuldades no início da vida sexual e depressão.^40^

Outros estudos demonstram haver um maior comprometimento nutricional nos pacientes de DC em relação àqueles com RCU, em consequência da maior gravidade e extensão da área acometida no trato digestório. Habitualmente a DC atinge segmentos do intestino delgado responsáveis pela absorção, além de frequentemente acometer o cólon e outras áreas do tubo digestivo.^35^^-^ 37 Não só a extensão do acometimento, mas também a gravidade, a forma de apresentação, as manifestações extraintestinais e comorbidades que podem comprometer o estado nutricional devem ser levadas em consideração.^34^^,^ 41 Se não identificada e tratada, a desnutrição em pacientes pediátricos pode causar graves problemas para a saúde, com piora do prognóstico e consequente redução da competência imunológica, aumento de infecções, retardo no crescimento e no desenvolvimento, problemas sociais e maiores taxas de comorbidades. A parada no crescimento e o retardo dos caracteres pubertários são manifestações da faixa pediátrica de importância significativa e podem ser os primeiros achados clínicos nesta condição, aspecto observado em várias publicações,^35^^-^ 37 enfatizando-se o retardo de crescimento relatado em 18 a 22% dos pacientes. Diversos estudos demonstram altas taxas de retardo de crescimento, muitas vezes antecedendo as manifestações intestinais, variando entre 5% e 88%, sendo que os pacientes com DC experimentaram maiores taxas de comprometimento nutricional quando comparados com os portadores de RCU. Em associação com o retardo de crescimento, há comprometimento do estado puberal em 20 a 30% dos pacientes, principalmente nos portadores de DC^38^^,^ 41 ^,^ 42 com causas multifatoriais.^35^^,^ 36 Outras explicações para o retardo do crescimento estão relacionadas ao uso de medicamentos, sobretudo corticoterapia e distúrbios hormonais causados tanto pelos efeitos diretos dos processos inflamatórios quanto pelo retardo da puberdade. Crianças com diagnostico precoce de DII nas fases pré-púbere ou púbere merecem atenção especial para o seu crescimento linear.^35^^,^ 37 ^,^ 42

Outro aspecto é a mudança no perfil de sobrepeso ou obesidade na população pediátrica com DII, com tendência ao aumento do número de pacientes com sobrepeso ou obesidade, variando de 9-34%, a depender do subtipo da doença, com maior prevalência em portadores de RCU. Em estudo realizado nos Estados Unidos, foi encontrado IMC elevado em 20 a 30% dos pacientes, estando os valores mais elevados nos portadores de RCU.^41^ Esse aumento dos índices de sobrepeso e obesidade em pacientes pediátricos com DII pode ser explicado, em parte, pela presente epidemia de síndrome metabólica que ocorre no mundo, devido às mudanças de hábitos alimentares.

Estudos demonstram que deficiências de minerais, oligoelementos e vitaminas são comuns em portadores de DII, sendo a deficiência de vitamina D a mais comum, chegando a 60% em alguns casos.^41^ Estas várias deficiências refletem uma perda crônica de sangue, diarreia crônica ou comprometimento de sítios específicos de absorção, além da má absorção difusa por conta da extensão da inflamação ou ressecções cirúrgicas.^34^ Em alguns estudos, a deficiência mais comum foi a de vitamina D nos pacientes com RCU, quando comparados aos com DC, diferentemente das publicações da literatura nas quais há maior prevalência nos portadores de DC.^38^^,^ 40

## Conclusões

Um numero limitado de artigos foi encontrado e não há estudos sobre o tema na América do Sul. Há alterações nutricionais de etiologia multifatorial nas doenças inflamatórias intestinais em pediatria, ressaltando-se parada no crescimento e desenvolvimento puberal, baixo peso e deficiências vitamínicas. Os comprometimentos nutricionais relatados foram mais expressivos nos pacientes portadores de Doença de Crohn, e o sobrepeso e obesidade mais frequentes na Retocolite Ulcerativa. A avaliação nutricional detalhada deve ser realizada periodicamente em todas as crianças e adolescentes portadores de doenças inflamatórias intestinais, combinada com diferentes métodos e recursos multidisciplinares. São necessários mais estudos na faixa etária pediátrica com populações mais numerosas.
